# The willingness to use internet-based home care services and its influencing factors among rural older people in Henan Province, China: a cross-sectional study

**DOI:** 10.3389/fpubh.2024.1399867

**Published:** 2024-08-13

**Authors:** Yuwen Yan, Yan Zhang, Huizhong Zhang, Xizheng Li, Lixue Meng, Yutong Tian

**Affiliations:** School of Nursing and Health, Zhengzhou University, Zhengzhou, Henan, China

**Keywords:** rural, older people, internet-based home care services, willingness to use, cross-sectional study

## Abstract

**Background:**

To meet the growing health needs of older people, the Chinese government has introduced internet-based home care services. However, most rural older people have not yet benefited from such services, and the willingness to use these services and the factors influencing them remain unclear.

**Objective:**

We aim to investigate the current willingness of rural older people to use internet-based home care services and analyze the factors.

**Methods:**

We conducted a cross-sectional study across multiple centers using general information and self-developed questionnaires. Qualitative interviews, a literature review and the Delphi method were employed to develop the questionnaire. A total score of the questionnaire above 78 or higher indicates a high willingness to use internet-based home care services. Descriptive statistics, *t*-tests, one-way ANOVA, and multiple linear regression were used to explore the factors that influence the willingness of rural older people to use internet-based home care services.

**Results:**

We surveyed 349 rural older people. The total score of rural older people’s willingness to use internet-based home care services was 84.49 ± 10.88, indicating high willingness, with the highest score for the dimension of perceived usefulness and lower scores for the dimension of perceived ease of use. Multiple linear regression analysis revealed that gender, education level, residence status, number of chronic diseases, and knowledge of internet-based home care services were the most important factors for rural older people (all *p* < 0.05).

**Conclusion:**

The willingness of rural older people to use internet-based home care services is high. Thus, it is recommended that county hospitals increase public awareness of such services, optimize the design of their interfaces, and support family and social resources from relevant departments that can maximize access, so as to provide a reference for later relevant departments to enhance willingness.

## 1 Introduction

China is one of the countries with the largest population of older people and the fastest aging rate (1). According to China’s National Bureau of Statistics, as of the end of 2021, China’s population aged 60 and above was 260 million, accounting for 18.70% of China’s total population, and 120 million people aged 60 and above were in rural areas, accounting for 23.81% of the total population in rural areas (2). The increase in the number of older people has led to a surge in demand for home care services (3). With the aging problem becoming increasingly serious, the medical service industry is facing major challenges (4), so how to meet the demand for care services for older people in rural areas needs urgent attention.

With the development of cloud computing, big data, artificial intelligence and other information technology, the application of the internet in home care services has become more promising (5). Internet-based home care services mean that nurses in medical institutions provide services for older adult and special people with mobility difficulties through information technology such as the internet (6). In 2019, the National Health Commission of the People’s Republic of China issued “the notice on the pilot work of internet-based home care services,” which expanded nursing services from institutions to communities and families (7). The notice clarified the main body, target population and items of internet-based home care service. The main body is composed of registered nurses with at least 5 years of clinical nursing experience and a technical title of nurse or above. The main target population is patients with relapsing illnesses, chronic illnesses, or older adult individuals. The services items included skin care, catheter maintenance, specimen collection, basic nursing care, rehabilitation care and hospice care.

In countries such as the United States (8), Canada (9) and Japan (10), home care has become an effective way to cope with aging. Studies have shown that the implementation of internet-based home care services has many benefits. First, the pressure and work intensity of clinical nurses can be reduced by expanding the supply of care services (11–13). Second, it helps older people obtain high-quality medical services outside the hospital (14) and reduces the admission rate of older people (15–18). In other words, this new type of service provides a new solution to the serious aging situation in China.

According to the data released by the China internet Network Information Center, as of December 2023, the internet penetration rate in China’s rural areas was 66.5%, and the number of rural internet users reached 326 million (19), which provides a foundation for the implementation of internet-based home care services. The willingness of older adult to use these services can directly affect the usage rate of internet-based home care services; however, domestic research on willingness to use these services has focused mainly on nurses (20, 21) and community older people (22, 23), and there are fewer studies targeting rural older adults. Compared with urban areas, rural areas are geographically remote, economically disadvantaged, and culturally and educationally backward, so the willingness of rural older people differs from that of community older people. Therefore, this study investigated the willingness of some rural older people to use internet-based home care services, and analyzed its influencing factors, in order to provide a reference for relevant departments to develop a service model suitable for rural older people.

## 2 Materials and methods

### 2.1 Study design and participants

This study used a multicenter and cross-sectional survey research design. From July to September 2022, a random number table method was used to select three prefectures from 17 prefectures in Henan Province, China, as the study site and subsequently to facilitate the selection of questionnaires from 26 administrative villages under them for rural older people. The inclusion criteria were as follows: (a) were aged more than 60 years and had lived in the countryside for more than 1 year, (b) had no serious visual–auditory or communication dysfunction, and (c) provided informed consent and voluntary participation in this study. The exclusion criteria were as follows: rural older people with severe mental illness or cognitive dysfunction who were unable to cooperate with the survey. We used an empirical method to calculate the sample size, which involved a total of 11 demographic variables and 5 variables related to willingness to use internet-based home care services in this study, taking 5–10 times the number of variables, and considering a 10–20% missed visit rate, the calculated sample size range was 89–200.

### 2.2 Measures

(1) General information questionnaire: a self-developed questionnaire, including gender, age, education level, marital status, number of children, residence status, main source of income, and individual monthly income.

(2) Questionnaire on the willingness to use internet-based home care services. The questionnaire was designed by the researcher on the basis of a literature review (23, 24), national health policies and in-depth interviews with rural older people. Two rounds of expert consultation were conducted for this study, with 15 experts, 9 in nursing, 2 in clinical medicine, 3 in public health, and 1 engineer in computer science and technology; there were 30 initial entries. After two rounds of expert consultation, a total of 9 entries were deleted, for example, “I think the practicing qualification of internet-based home care services should be clarified before booking the service”; 5 entries were added, for example, “I think that internet-based home care services can save my traveling expenses to and from hospitals”; and three entries that are broad or improperly expressed, for example, item 1, “I think that internet-based home care services can reduce the time needed to go to the hospital,” were adjusted to “I think that internet-based home care services make my life more convenient.” A convenience sampling method was used to recruit 30 rural older adults for presurvey and cognitive interviews to assess the concepts, semantics, and content of the scales. There were no specific modifications, and the average completion time was typically 5–10 min.

A total of 237 rural older people voluntarily completed the questionnaire for the purpose of exploratory factor analysis and calculation of Cronbach’s coefficient. The five common factors of willingness to use internet-based home care services were extracted, and the cumulative contribution rate of variance reached 65.143%. The loadings of each corresponding common factor were greater than 0.5 and greater than the loadings of the other factors, thus achieving equivalence between the scale structure and the theory.

The questionnaire consists of five dimensions: perceived usefulness (7 items, Cronbach’s *α* = 0.853, range 0–35 points), perceived ease of use (3 items, Cronbach’s *α* = 0.977, range 0–15 points), subjective norms (4 items, Cronbach’s *α* = 0.826, range 0–20 points), perceived risk (8 items, Cronbach’s *α* = 0.841, range 0–40 points), willingness to use (4 items, Cronbach’s *α* = 0.944, range 0–20 points) and total scale (26 items, *α* = 0.841, range 0–130 points). The perceived risk dimension is reverse scored. Likert 6-level scoring was adopted for all items in this section, with scores ranging from 0 to 5 (from “strongly reluctant” to “strongly willing”). A score of 78 or above indicates a high willingness to use internet-based home care services; a score below 78 indicates a low willingness to use internet-based home care services.

### 2.3 Data collection

The researcher contacted the person in charge of a township health center in Henan Province before the survey, followed the health center’s medical examination team to conduct an in-depth survey in the rural areas, explained the purpose and significance of the survey before the survey, and obtained informed consent to be distributed on the spot, and all the questionnaires were anonymous; when the older people had difficulties reading the questionnaires, the researcher held a neutral attitude toward the older people reading the questionnaire entries and assisted in ticking the boxes, and the questionnaires were recovered on the spot. A total of 370 questionnaires were distributed, and 349 valid questionnaires were recovered, for a valid recovery rate of 94.32%.

### 2.4 Statistical analysis

SPSS 25.0 software was used for statistical analysis of the data. We described categorical data as absolute frequencies and percentages and continuous data as the mean and standard deviation (SD). The categorical data are expressed as frequencies and percentages (%), and the continuous data are expressed as the means and standard deviations (SDs); independent sample t tests and one-way ANOVA were used for comparisons between groups, and multiple linear regression analysis was used for influencing factor analysis. *p* < 0.05 indicated that the difference was statistically significant.

## 3 Results

### 3.1 Participants’ characteristics

A total of 349 rural older people were investigated in this study, with an average age of 70.91 ± 5.93 years; 122 (35.0%) were male, and 227 (65.0%) were female. The remaining general information is shown in Table 1.

**Table 1:** Intergroup comparison of the willingness to use internet-based home care services of rural older people (*n* = 349).

Variables	*N* (%)	Mean ± SD	*F/t*	*P*
Gender			3.749^a^	<0.001
Male	122 (35.0)	87.42 ± 12.22		
Female	227 (65.0)	82.92 ± 9.76		
Age (years)			1.714^b^	0.182
60–69	156 (44.7)	85.44 ± 10.93		
70–79	162 (46.4)	83.34 ± 11.22		
≥80	31 (8.9)	85.74 ± 8.20		
Education level			7.453^b^	<0.001
Illiteracy	169 (48.4)	81.94 ± 9.69		
Primary school	87 (24.9)	85.80 ± 11.88		
Middle school	74 (21.2)	87.20 ± 10.65		
High school and above	19 (5.5)	90.63 ± 11.76		
Marital status			1.606^a^	0.109
Married	249 (71.3)	85.08 ± 10.88		
Widowed	100 (28.7)	83.02 ± 10.78		
Number of Children			0.049^b^	0.952
1	21 (6.0)	84.90 ± 9.58		
2	140 (40.1)	84.29 ± 10.82		
≥3	188 (53.9)	84.60 ± 11.11		
Residence Status			3.126^b^	0.045
Living with children	59 (16.9)	81.88 ± 11.05		
Living with spouse	230 (65.9)	84.55 ± 10.35		
Live alone	60 (17.2)	86.83 ± 12.27		
Source of income			1.882^b^	0.113
Retirement pay	33 (9.5)	87.45 ± 10.88		
Pensions	43 (12.3)	83.42 ± 11.24		
Child allowance	98 (28.1)	86.01 ± 10.80		
Farming	139 (39.8)	82.99 ± 11.13		
Part-time job	36 (10.3)	84.75 ± 8.95		
Individual monthly income/Yuan			1.020^b^	0.384
0 ~ 499	43 (12.3)	84.37 ± 10.81		
500 ~ 999	214 (61.3)	84.00 ± 11.41		
1,000 ~ 1,499	58 (16.6)	84.66 ± 8.77		
1,500~	34 (9.8)	87.50 ± 10.72		
Number of chronic diseases			2.745^b^	0.043
None	78 (22.3)	83.44 ± 9.68		
1^c^	95 (27.2)	82.54 ± 10.33		
2^c^	96 (27.5)	85.32 ± 10.62		
≥3^c^	80 (23.0)	86.85 ± 12.47		
Whether they knew about internet-based home care services			−4.095^a^	<0.001
No	299 (85.7)	83.54 ± 10.41		
Yes	50 (14.3)	90.20 ± 11.98		

a is *t* value; b is *F* value; c is the measured disease type, including hypertension, coronary heart disease, diabetes, stroke, hyperthyroidism, etc.

### 3.2 The scores of the willingness to use internet-based home care services of rural older people

**Figure 1:**
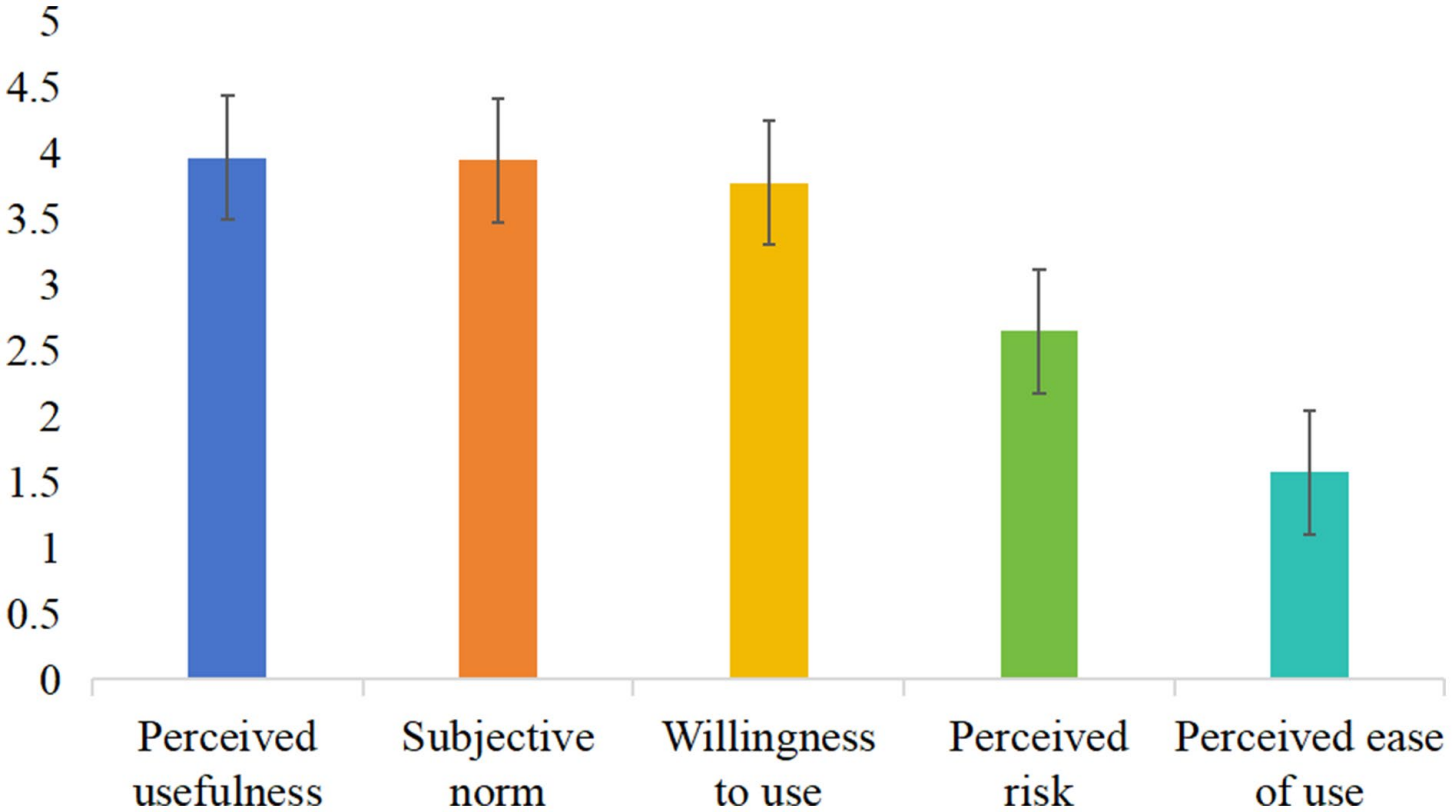
Average scores of entries in the dimension of willingness to use internet-based home care services of rural older people. The horizontal axis indicated the different dimensions of the questionnaire on the willingness to use internet-based home care services, and the vertical axis indicated the average scores of entries in the different dimensions.

**Figure 2:**
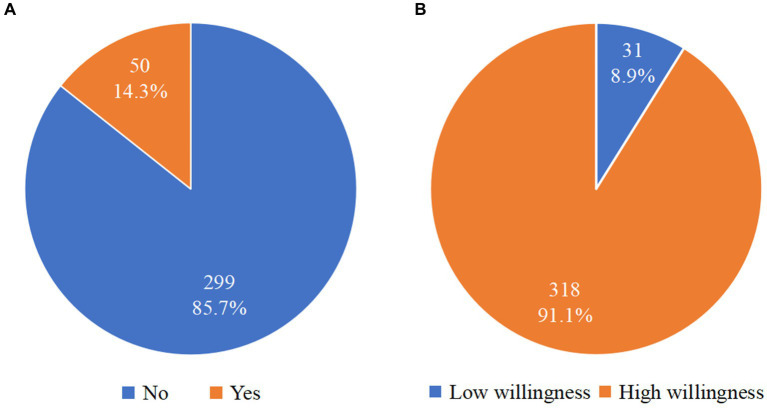
Awareness and willingness of rural older people to use internet-based home care services. **(A)** Whether rural older people knew about internet-based home care services. **(B)** Distribution of high and low willingness to use internet-based home care services among rural older people.The numbers on the pie chart represent the number and percentage of people who chose the corresponding option.

The total score of the 349 rural older people who were willing to use internet-based home care services was 84.49 ± 10.88, and the total average score was 3.24 ± 0.41. See Table 2 and Figure 1 for details. Most rural older people (91.1%) were willing to use internet-based home care services (Figure 2).

**Table 2:** Scores for items on dimensions of willingness to use internet-based home care services of rural older people (*n* = 349).

Willingness to use internet-based home care services	Total scores of entries in the dimension	Average scores of entries in the dimension
Perceived usefulness	27.74 ± 5.05	3.96 ± 0.72
Subjective norm	15.76 ± 2.78	3.94 ± 0.69
Willingness to use	15.08 ± 2.83	3.77 ± 0.70
Perceived risk	21.18 ± 5.90	2.64 ± 0.73
Perceived ease of use	4.73 ± 2.67	1.57 ± 0.89
Totals	84.49 ± 10.88	3.24 ± 0.41

Totals, total score for the whole scale.

### 3.3 Factors influencing rural older people’s willingness to use internet-based home care services

According to the general information of the survey respondents, the scores of rural older people’s willingness to use internet-based home care services were analyzed by using independent samples t tests or one-way ANOVA. The results of the study showed that the differences in gender, education level, residence status, number of chronic diseases, and knowledge of internet-based home care services were statistically significant (*p* < 0.05). See Table 1 for details.

### 3.4 Multiple linear regression analyses of rural older people’s willingness to use internet-based home care services

Taking the score of rural older people’s willingness to use internet-based home care services as the dependent variable, the statistically significant variables in the one-way ANOVA were included in the multiple linear regression equation. The independent variable assignments are shown in Table 3. The results show that gender, education level, residence status, number of chronic diseases, and knowledge of internet-based home care services are factors influencing the willingness of rural older people to use internet-based home care services (*p* < 0.05), as shown in Table 4.

**Table 3:** Assignment methods of independent variables.

Independent variable	Description of the assignment
Gender	Male = 1, Female = 2
Education level	Illiteracy = 1, Primary school = 2, Middle school = 3, High school and above = 4
Residence status	Dummy variable for “live alone “as control group: Live alone (*Z*_1_ = 0, *Z*_2_ = 0), Living with children (*Z*_1_ = 1, *Z*_2_ = 0), Living with spouse (*Z*_1_ = 0, *Z*_2_ = 1)
Number of chronic diseases	None = 1, 1 type = 2, 2 types = 3, ≥3 types = 4
Whether they knew about internet-based home care services	No = 0, Yes = 1

Residential status: as an unordered multicategory variable that requires a dummy variable before entering the regression equation.

**Table 4:** Multiple linear regression analysis results on rural older people’s willingness to use internet-based home care services.

Independent variable	Regression coefficient	Standard error	Standardized regression coefficient	*t*	*P*
(Constant)	77.540	3.685		19.797	<0.001
Gender	−2.712	1.268	−0.119	−2.139	0.033
Education level	2.374	0.645	0.206	3.683	<0.001
Residence status (compared to live alone)					
Living with spouse	−3.500	1.488	−0.153	−2.353	0.019
Living with children	−4.593	1.870	−0.158	−2.456	0.015
Number of chronic diseases	1.084	0.514	0.107	2.112	0.035
Whether they knew about internet-based home care services	6.498	1.564	0.209	4.154	<0.001

## 4 Discussion

### 4.1 Rural older people’s willingness to use internet-based home care services is high

The results of this study show that the total willingness score of rural older people was 84.49 ± 10.88, indicating a high willingness to use such services. The perceived ease of use dimension scored the lowest, with a dimension score of 4.73 ± 2.67. This may be because the age of the survey respondents was high, the average age was 70.91 ± 5.93 years, the education level was poor, 48.4% of the older people had not attended primary school, and the majority of the older people were equipped with smart devices by their children, but the older people were not able to use them (25, 26). It was more difficult for older people to place orders online, which affected their willingness. Therefore, it is recommended that service providers fully mobilize the family and social resources of rural older people (27, 28), helping them cross the digital divide and reduce technological anxiety. At the same time, it is recommended that county hospitals develop multichannel booking methods such as online, on-site, and phone booking methods; optimize the interface design and functions of internet-based home care service platforms (e.g., reduce application options, increase page icons and fonts) (29, 30); take into full consideration the habits of older people; simplify the online booking process (31); and provide older people with voice guidance and manual consultation, so as to improve the user experience of rural older people and subsequently increase the willingness of rural older people.

### 4.2 Analysis of factors influencing rural older people’s willingness to use internet-based home care services

#### 4.2.1 Gender

This study revealed that the willingness of older adult women was lower than that of men (*p* < 0.05). The reason is that older adult women attach great importance to spending money because of their lack of income sources and hard experiences in their early years, they are thriftier than men, and they pay special attention to the price of services before using services. At present, the price of internet-based home care services mainly consists of service fees, transportation fees, and one-time medical consumable fees, and the comprehensive cost is relatively high. A high price makes older adult women more inclined to choose offline medical institutions for treatment and nursing. To better promote internet-based home care services, relevant departments should improve the medical insurance system, reduce the financial burden of the older adult, so that more rural older people can afford to pay for the program. At the same time, we should learn from the advanced experience of the nursing insurance system, family ward and long-term care system established by foreign countries (32), effectively combine government funding, medical insurance and personal payment by residents, and continuously improve price policy.

#### 4.2.2 Education level

The study revealed that the greater the education level was, the greater the willingness of rural older people to use internet-based home care services (*p* < 0.05). The reason for this is that rural older people with a high education level have greater acceptance of new things and greater ability to learn and master new things (33), can obtain information related to internet-based home care services through multiple channels (such as mobile phones, books, newspapers, bulletin boards, broadcasting, etc.), and are more willing to try internet-based home care services. It is recommended that county hospitals contact rural doctors in their districts and use rural doctors as a medium (34), focusing on the rural older adult population in the district. After gaining an in-depth understanding of their needs for home-based services, they are promptly connected with the county hospital service staff and guided in the use of the service.

#### 4.2.3 Residence status

This study revealed that residence status is an influential factor for the willingness of rural older people to use internet-based home care services (*p* < 0.05). Older people living alone are more willing to use services than those living with their children and spouses, similar to the results obtained by Ma et al. (35). The reason is that compared with those living in other states, rural older people living alone can use relatively limited external resources for older adult care. When facing health problems, older people living alone are able to conveniently and quickly book nursing services through online platforms, which prevents them from being able to seek timely medical care due to inconvenient transportation or physical inconvenience. However, relevant studies have shown that the quality of current internet-based home care services varies (23); therefore, further strengthening the management system and service specifications of service personnel, enhancing the professional knowledge and skills of nurses, and optimizing the quality of nurse services are recommended.

#### 4.2.4 Number of chronic diseases

This study revealed that rural older people with more chronic diseases were more willing to use internet-based home care services (*p* < 0.05). The reason for this may be that rural older people with more chronic diseases have poorer health conditions, and as their age and condition progress, their demand for home care increases, so their willingness to use internet-based home care services increases (5, 14, 36). This suggests that we should focus on the needs of rural older people with chronic diseases at different levels and provide them with accurate and personalized nursing services based on their needs, thus increasing their willingness.

#### 4.2.5 Whether they knew about internet-based home care services

This study revealed that rural older people who have some knowledge of internet-based home care services are more willing to use such services, which is similar to the findings of Liu et al. (23). The reason for this is that rural older people who have a certain understanding of internet-based home care services can better understand the accessibility and convenience of services (37). When encountering health problems, they are more inclined to use internet-based home care services to quickly communicate with healthcare personnel (38) and truly realize medical status without leaving home, so rural older people are willing to use these services. It is suggested that we increase the publicity of internet-based home care services, which can be promoted by combining online (TV, radio, etc.) and offline (large screen display, leaflet distribution, etc.) services. Second, we can choose “star older people” who have used internet-based home care services in the same village to share their experiences, which will increase rural older people’s awareness and make them fully aware of the advantages of the services, thus increasing their willingness to use internet-based home care services.

## 5 Conclusion

The results of this study show that rural older people’s willingness to use internet-based home care services is high, and their perceived ease of use is low. Gender, education level, residence status, number of chronic diseases, and knowledge of internet-based home care services are the factors influencing the willingness of rural older people. It is recommended that county hospitals increase the publicity of internet-based home care services to improve the service knowledge rate of rural older people. In addition, relevant medical institutions or platforms should pay attention to the convenience and friendly design of reservation systems to optimize the service reservation process and at the same time increase the willingness of children to feed back by connecting with their children. When older adult users encounter a situation that they cannot dispose of, they can connect their children with one key to help them complete the operation, so as to increase the willingness of the rural older adult to use the internet home care service and provide a reference for later relevant departments to develop an internet home care service model suitable for the rural older adult.

## 6 Limitations

Due to time and space constraints, this study selected only rural older people in Henan Province, which is not representative of the overall population of older people in rural areas. It is recommended that a large-scale, multicenter collaborative study be conducted in the future to make the research results more representative. In addition, this study did not investigate the specific service needs of rural older people, and in the future, it is recommended that a combination of quantitative and qualitative research methods be used to analyze in depth the needs of rural older people for internet-based home care services to enrich the content and results of the study.

## Data availability statement

The original contributions presented in the study are included in the article/supplementary material, further inquiries can be directed to the corresponding author.

## Ethics statement

The study was approved by the Life Sciences Ethics Review Committee of Zhengzhou University (ZZUIRB2021-155). This study was conducted in accordance with the local legislation and institutional requirements. The participants provided their written informed consent to participate in this study.

## Author contributions

YWY: Data curation, Formal analysis, Investigation, Writing – original draft, Writing – review & editing. YZ: Conceptualization, Funding acquisition, Methodology, Resources, Writing – review & editing. HZZ: Formal analysis, Investigation, Visualization, Writing – review & editing. XZL: Data curation, Investigation, Writing – review & editing. LXM: Data curation, Formal analysis, Writing – review & editing. YTT: Formal analysis, Investigation, Writing – review & editing.
